# Supplementary material for: Investigating the influence of physiologically relevant hydrostatic pressure on CHO cell batch culture
Source: Sci Rep. 2021 Jan 8;11:162. doi: 10.1038/s41598-020-80576-8 (PMC7794228; doi:10.1038/s41598-020-80576-8)
Supplement: Supplementary file 1 — Supplementary Information 1. [file 41598_2020_80576_MOESM1_ESM.zip › S1/IgG/Feb17 60 90 standard/standard/0ugml.pdf]

===

Acq. Operator : SYSTEM Seq. Line : 1
Sample Operator : SYSTEM
Acq. Instrument : HPLC-0039 Location : 1
Injection Date : 2/17/2019 5:22:17 PM Inj : 1
Inj Volume : 100.000 µl
Different Inj Volume from Sample Entry! Actual Inj Volume : 50.000 µl
Sequence File : C:\Users\Public\Documents\ChemStation\1\Data\TK_CHO_IgG 2019-02-17 17-20-57
\TK_CHO_IgG.S
Method : C:\Users\Public\Documents\ChemStation\1\Data\TK_CHO_IgG 2019-02-17 17-20-57
\CHO_IgG_LC.M (Sequence Method)
Last changed : 2/8/2019 3:17:23 PM by SYSTEM

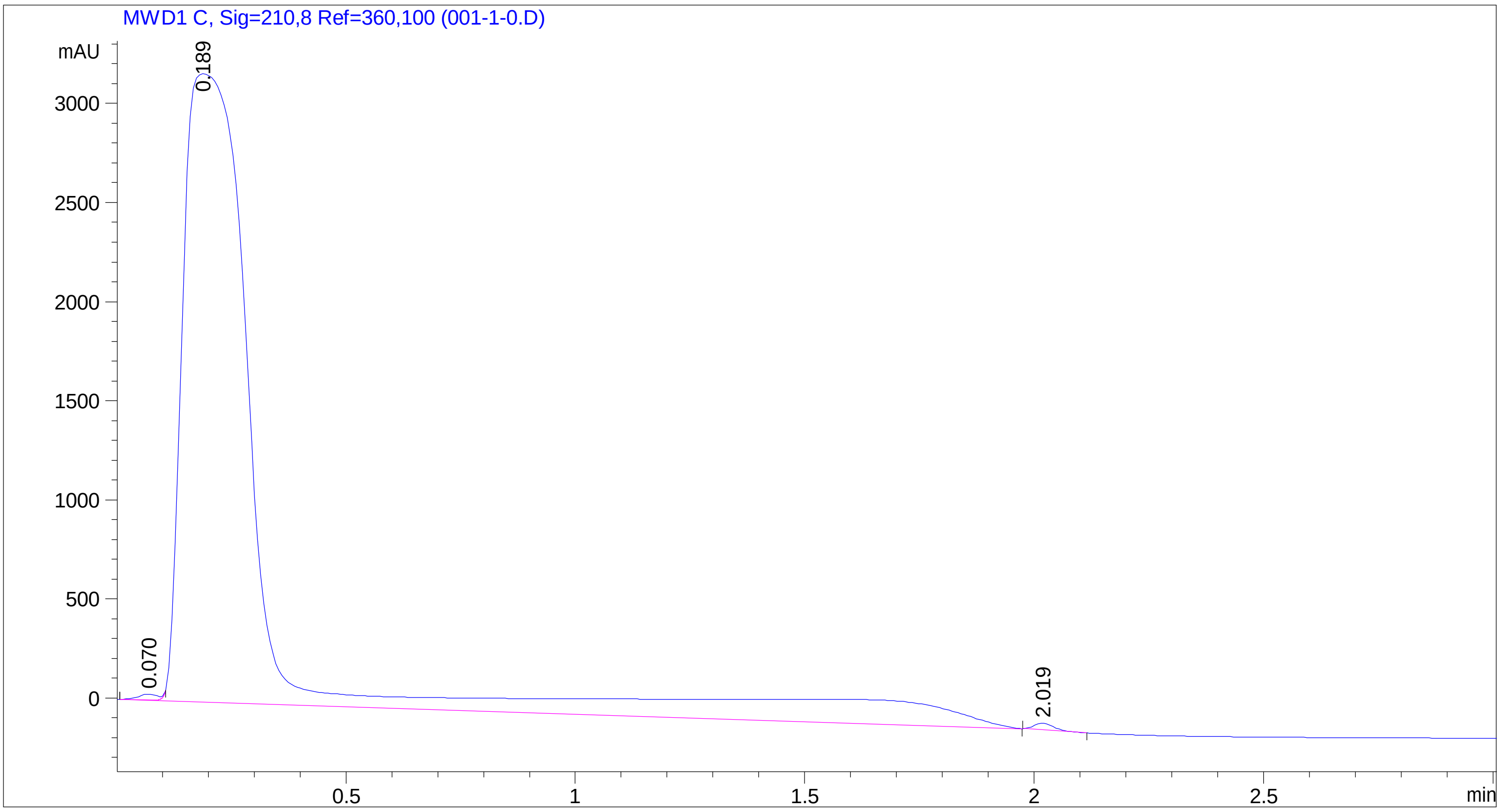

===

Area Percent Report

===

Sorted By : Signal
Multiplier : 1.0000
Dilution : 1.0000
Use Multiplier & Dilution Factor with ISTDs

Signal 1: MWD1 C, Sig=210,8 Ref=360,100

Peak #	RetTime [min]	Type	Width [min]	Area [mAU*s]	Height [mAU]	Area %
1	0.070	BV E	0.0454	84.53372	29.98203	0.2301
2	0.189	VB R	0.1786	3.65574e4	3168.05811	99.4955
3	2.019	BBA	0.0465	100.84453	34.11286	0.2745

Totals : 3.67428e4 3232.15300

===

*** End of Report ***
